# Leukemia Cutis at Initial Diagnosis of Acute Myeloid Leukemia Consistent With Therapy‐Related Disease After Lung Cancer Chemotherapy: A Case Report

**DOI:** 10.1155/crh/1198642

**Published:** 2026-05-09

**Authors:** Adam Bowen, Nicholas DiLoreto, Maha Bayya, Robert McDonald, Daniel Isaac

**Affiliations:** ^1^ Division of Hematology/Oncology, Karmanos Cancer Institute at Mclaren Greater Lansing Hospital, Lansing, Michigan, USA; ^2^ Department of Pathology, Mclaren Greater Lansing Hospital, Lansing, Michigan, USA, mclaren.org

**Keywords:** DNMT3A, leukemia cutis, non–small-cell lung cancer, PPM1D, therapy-related AML, U2AF1

## Abstract

**Introduction:**

Leukemia cutis (LC) is characterized by dermal infiltration of leukemic blasts and typically occurs in patients with known leukemia. Rarely, LC is identified at the time of initial diagnosis of systemic disease, especially acute myeloid leukemia (AML). We describe a case of LC identified at the initial diagnosis of AML consistent with therapy‐related disease in a patient with metastatic non–small‐cell lung cancer (NSCLC) previously treated with cytotoxic chemotherapy and immunotherapy.

**Case Presentation:**

A 78‐year‐old woman with Stage IV NSCLC (adenocarcinoma, no actionable mutations, PD‐L1 negative) initially received carboplatin, pemetrexed, and pembrolizumab, complicated by neutropenic fever. She later enrolled in the SHERLOC trial (MM‐121 + docetaxel), discontinued due to colitis and neutropenia. Maintenance therapy with pembrolizumab was continued intermittently alongside stereotactic body radiation and cryoablation. After several years of disease control, follow‐up PET/CT demonstrated new adrenal and iliac bone lesions concerning for progression. Shortly thereafter, she developed multiple pruritic, erythematous plaques on her extremities, accompanied by fatigue, weight loss, and cytopenias. Laboratory evaluation showed leukocytosis, macrocytic anemia, and severe thrombocytopenia, with peripheral smear revealing 72% circulating blasts. Punch biopsy confirmed LC, demonstrating dermal infiltration by myeloid blasts (CD4, CD43, CD68, and MPO positive). Flow cytometry and bone marrow biopsy established AML with > 75% myeloblasts. Next‐generation sequencing identified DNMT3A, U2AF1, and PPM1D mutations. Conventional cytogenetic analysis was noninformative because no metaphase cells were obtained, AML FISH was normal, and PML‐RARA FISH was negative, findings consistent with therapy‐related AML.

**Interventions and Outcomes:**

The patient received hydroxyurea, transfusion support, and tumor lysis prophylaxis. Given her age, ECOG 1, adverse‐risk mutations, and personal preference, she declined further chemotherapy and transitioned to hospice.

**Conclusion:**

LC at the time of initial AML diagnosis is uncommon and portends poor prognosis. Clinicians should remain vigilant for new skin lesions and cytopenias in cancer survivors previously exposed to chemotherapy, as early recognition of extramedullary AML may expedite timely diagnosis and care planning.

## 1. Introduction

Leukemia cutis (LC) refers to infiltration of the skin by neoplastic leukocytes, most commonly in chronic lymphocytic leukemia (CLL), acute myeloid leukemia (AML), and T‐cell lineages. While most cases occur in patients with known leukemia, primary manifestation preceding systemic disease is uncommon, with about 23%–44% of cases preceding leukemia diagnosis [1–3]. The pathogenesis of LC is thought to involve chemokine receptors, integrins, and adhesion molecules promoting skin homing. Clinically, LC can present as papules, nodules, macules, plaques, or ulcers, often on the trunk, extremities, and face. LC frequently indicates advanced disease and poor prognosis [1]. Risk factors for leukemogenesis include exposure to benzene, ionizing radiation, alkylating agents, and viruses [2]. Because LC can mimic other inflammatory or infectious dermatoses, biopsy and appropriate immunohistochemistry and, when possible, molecular profiling are essential for diagnosis [3].

## 2. Case Presentation

### 2.1. Patient Information

A 78‐year‐old woman with Stage IV non–small‐cell lung adenocarcinoma (NSCLC, T2N1M1b) involving the adrenal gland and hilar nodes and comorbid hypertension, COPD, hyperlipidemia, and depression presented to the emergency department with new pruritic skin lesions and malaise. Her family history was notable for cancer in her father and sister. She denied alcohol, vaping, and illicit drug use but reported ongoing tobacco use, variably documented as fewer than four cigarettes daily in routine screening versus one pack per day at the time of presentation. She followed a regular diet and reported no psychosocial risk factors or safety concerns at home. Throughout her oncologic course, tumor profiling consistently showed no actionable mutations (EGFR, ALK, ROS1, and BRAF) and absent PD‐L1 expression.

### 2.2. Clinical Findings

**FIGURE 1:**
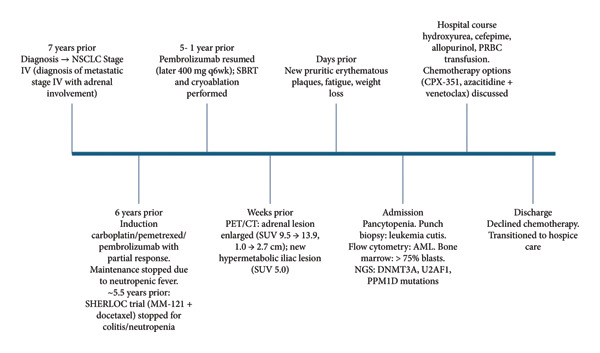
Timeline of clinical course and key events. Timeline of oncologic course, systemic therapies, and key diagnostic milestones.

Her oncologic treatment history spanned multiple systemic therapies. She initially received carboplatin (AUC 5 IV q3wk), pemetrexed (500 mg/m^2^ IV q3wk), and pembrolizumab (200 mg IV q3wk), achieving a partial response after four cycles. Maintenance pemetrexed and pembrolizumab were discontinued after neutropenic fever. She then enrolled in the SHERLOC trial (MM‐121 plus docetaxel 75 mg/m^2^ IV q3wk), discontinued after one cycle for colitis and neutropenia. Pembrolizumab monotherapy was resumed (200 mg IV q3wk, later transitioned to 400 mg IV q6wk). Local control included stereotactic body radiation therapy (6000 cGy in four fractions) and CT‐guided cryoablation. A summary of the patient’s disease course, diagnostic milestones, and key interventions is shown in timeline (Figure 1).

After several years of relative disease control, surveillance PET/CT demonstrated progression with a new hypermetabolic iliac bone lesion (SUV 5.0) and interval enlargement of the right adrenal metastasis from 1.0 cm (SUV 9.5) to 2.7 cm (SUV 13.9) (Figures 2(a), 2(b), and 2(c)). Within days, she developed multiple nontender, intensely pruritic plaques on the upper extremities, with additional lesions on the right foot and left ear, which had been present for approximately 2 days before presentation. The most striking lesion was a 2.5–3‐cm erythematous, indurated plaque with superficial breakdown on the right upper arm with two similar lesions on the left arm (Figures 3(a), 3(b), and 3(c)). She also reported fatigue, diarrhea without blood for 1.5 weeks, poor oral intake, urinary incontinence, and unintentional weight loss. On admission, she was afebrile (36.9°C), with a heart rate of 88 bpm, blood pressure of 126/61 mmHg, respiratory rate of 16/min, and SpO_2_ of 97%. Exam revealed expiratory wheezing but otherwise normal cardiopulmonary and abdominal findings, no hepatosplenomegaly, and no lymphadenopathy.

FIGURE 2(a) Axial CT of the abdomen demonstrating right adrenal metastasis (arrow). Enlarging right adrenal lesion consistent with progressive metastatic non–small‐cell lung cancer. (b) PET/CT demonstrating hypermetabolic right adrenal lesion (arrow). Intense FDG uptake in the enlarging right adrenal metastasis (SUVmax 13.9, previously 9.5). (c) PET/CT demonstrating new hypermetabolic iliac lesion (arrow). Focal FDG uptake in the right iliac bone (SUVmax 5.0), consistent with new osseous metastasis.

**Figure:**
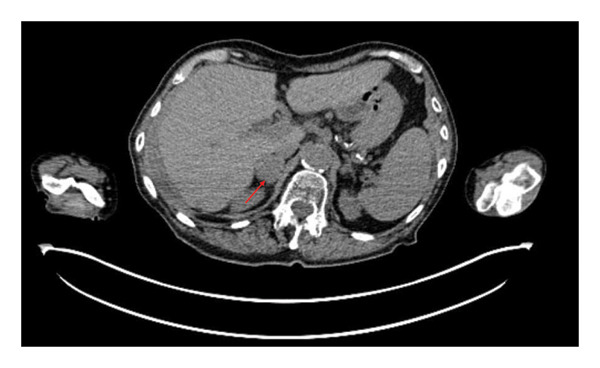
(a)

**Figure:**
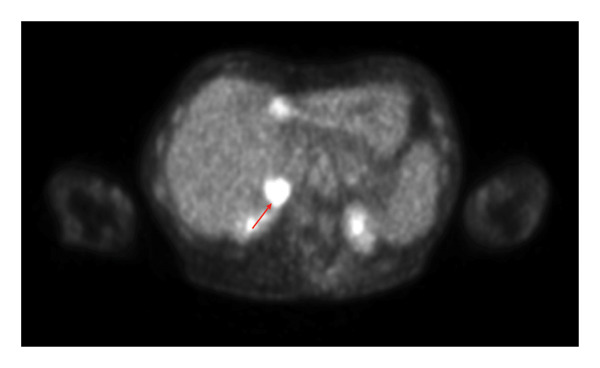
(b)

**Figure:**
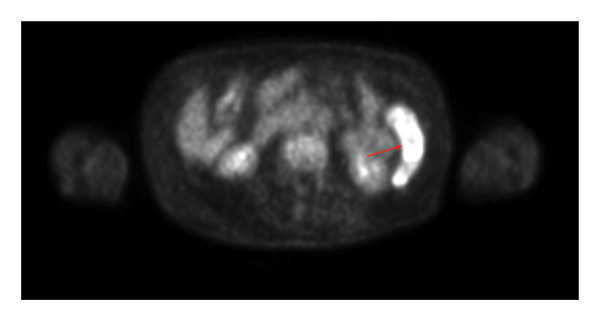
(c)

FIGURE 3(a) Clinical photograph of the right upper extremity lesion. Erythematous indurated plaque with overlying breakdown at the biopsy site on the right arm, consistent with leukemia cutis. (b) Clinical photograph of cutaneous lesion on the forearm. Solitary erythematous indurated plaque with central breakdown on the left forearm, consistent with leukemia cutis. (c) Clinical photograph of left cubital fossa lesion. Erythematous indurated plaque with central ulceration on the medial aspect of the left cubital fossa, consistent with leukemia cutis.

**Figure:**
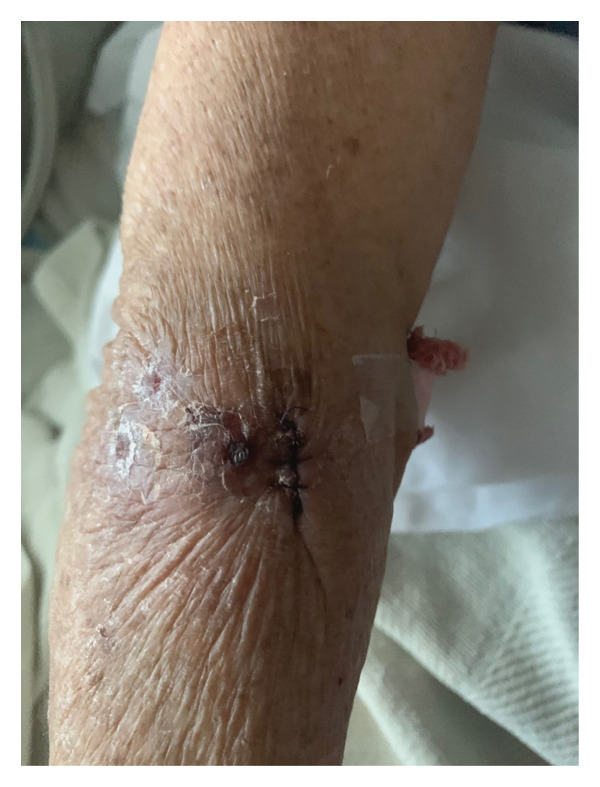
(a)

**Figure:**
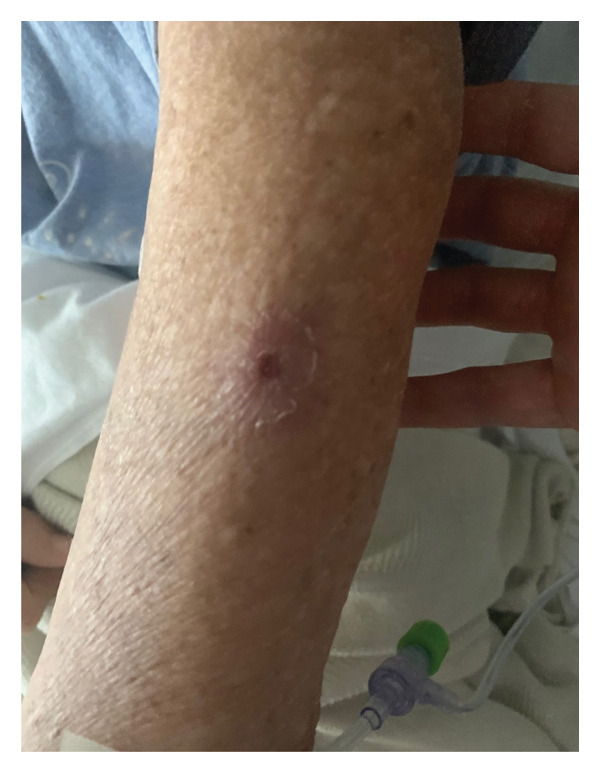
(b)

**Figure:**
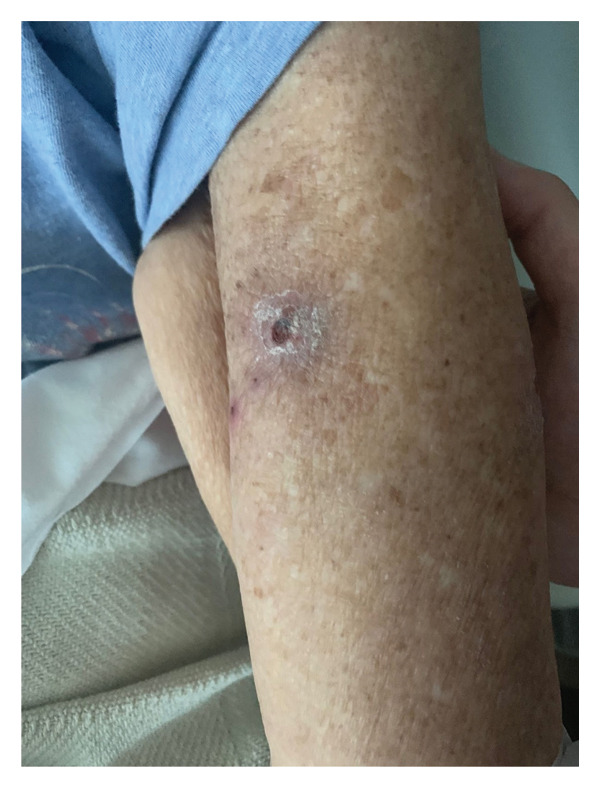
(c)

### 2.3. Diagnostic Assessment

Initial laboratory evaluation on presentation showed leukocytosis (WBC: 27.1–40.7 × 10^3^/μL), macrocytic anemia (hemoglobin: 8.3–9.4 g/dL, MCV > 103 fL), and severe thrombocytopenia (platelets: 40–45 × 10^3^/μL), in contrast to her baseline leukopenia two weeks earlier (Table 1).

**TABLE 1:** Peripheral blood counts over time relative to presentation.

Interval relative to presentation	WBC (× 10^3^/μL)	RBC (× 10^6^/μL)	Platelets (× 10^3^/μL)
∼5 months prior	3.78	4.04	163
∼4 months prior	2.94	3.64	148
∼3 months prior	2.54	3.41	133
∼3 weeks prior	2.04	2.93	83
∼2 weeks prior	2.97	2.92	75
∼1 week prior	27.17	2.68	45
Day of admission (Hospital Day 0)	40.72	2.41	40
Hospital Day 1	45.18	2.22	27
Hospital Day 2	48.42	2.03	22
Hospital Day 3	17.71	2.00	15

*Note:* Trend of white blood cells (WBC), red blood cells (RBC), and platelets over several months prior to admission and throughout hospitalization. The data highlight the transition from baseline leukopenia to marked leukocytosis with progressive anemia and thrombocytopenia, consistent with acute myeloid leukemia.

**FIGURE 5:**
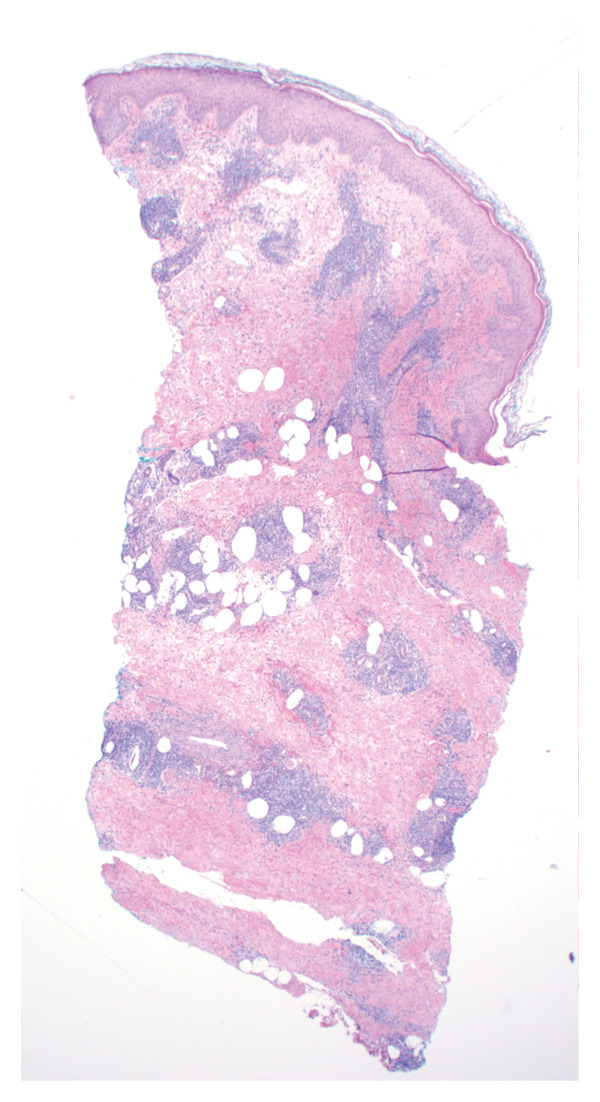
Punch biopsy of skin lesion demonstrating mononuclear infiltrate (hematoxylin and eosin stain). Diffuse dermal infiltration by atypical mononuclear cells; immunohistochemistry showed CD3 negativity with CD4 and CD34 positivity, consistent with leukemia cutis.

Bone marrow biopsy with smear demonstrated numerous blasts with prominent nucleoli within a hypercellular marrow (Figures 4(a) and 4(b)). Punch biopsy of the right arm lesion revealed dermal infiltration by atypical hematopoietic blasts positive for CD4, CD43, CD68, and MPO, consistent with LC (Figure 5). Flow cytometry demonstrated ∼62% blasts coexpressing CD34, CD117, CD13, CD33, and MPO, with CD15 positivity, partial TdT expression, and absent HLA‐DR, confirming AML (Tables 2 and 3). CD34 and CD117 supported an immature blast population, and MPO confirmed myeloid lineage. Given the low HLA‐DR expression, acute promyelocytic leukemia was considered, but the absence of Auer rods and negative PML‐RARA FISH argued against APL. Bone marrow biopsy revealed 75%–80% myeloblasts with irregular nuclear contours, open chromatin, and prominent nucleoli, without Auer rods. Reticulin staining showed mild fibrosis (MF1/3), and iron stain showed increased storage iron. Immunohistochemistry confirmed CD34+ blasts (85%–90%) with strong MPO expression. Conventional cytogenetic analysis was noninformative because no metaphase cells were obtained, and AML FISH was normal. Next‐generation sequencing identified DNMT3A, U2AF1, and PPM1D mutations, findings consistent with therapy‐related AML and adverse prognosis.

FIGURE 4(a) Bone marrow biopsy demonstrating acute myeloid leukemia. Hematoxylin and eosin–stained section showing markedly hypercellular marrow with diffuse blast infiltration, consistent with acute myeloid leukemia (> 75% blasts). (b) Bone marrow aspirate smear with increased blast forms (Wright–Giemsa stain). Bone marrow aspirate smear showing a markedly increased population of blasts with high nuclear‐to‐cytoplasmic ratio, open chromatin, and prominent nucleoli.

**Figure:**
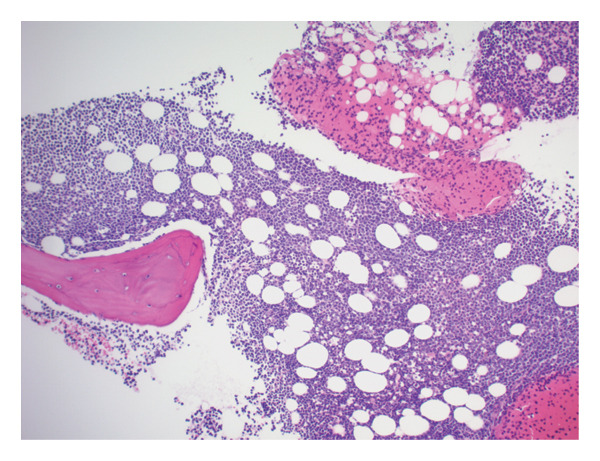
(a)

**Figure:**
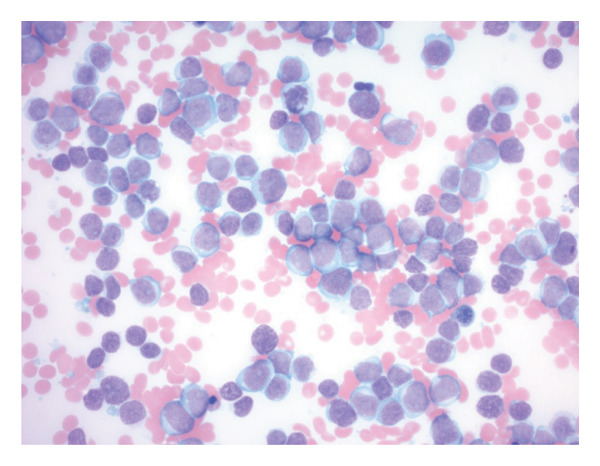
(b)

**TABLE 2:** Flow cytometry immunophenotyping of peripheral blood.

Antigen (marker)	% positive cells	Lineage/interpretation
CD45	98.1%	Pan‐leukocyte marker
CD1a	0.0%	Thymic precursor/immature dendritic cells
CD2	1.4%	T‐cell marker
CD3	0.1%	Mature T‐cell marker
CD4	79.2%	T‐helper cells/monocytes
CD4+/CD3+	0.2%	Helper T‐lymphocytes
CD5	0.1%	T‐cells/subset of B‐cells
CD7	1.6%	Early T/NK cell marker
CD8+/CD3+	0.0%	Cytotoxic T‐lymphocytes
CD10	0.4%	Pre‐B cells/granulocyte precursors
CD11b	20.1%	Granulocytes, monocytes, NK cells
CD11c	12.8%	Monocytes/dendritic cells
CD13	94.2%	Myeloid lineage marker
CD14	1.3%	Monocytes
CD15	59.2%	Granulocytes/eosinophils/monocytes
CD19	0.4%	B‐lymphocytes
CD33	41.9%	Myeloid lineage marker
CD34	95.5%	Hematopoietic stem/progenitor cells
CD41	7.0%	Platelets/megakaryocytes
CD61	10.6%	Platelets/megakaryocytes
CD117 (c‐Kit)	31.8%	Myeloid progenitors/stem cells
CD235a (glycophorin A)	0.4%	Erythroid lineage marker
HLA‐DR	0.9%	Antigen‐presenting cells/activated blasts
Kappa/CD19+	0.0%	B‐cell light chain—kappa
Lambda/CD19+	0.0%	B‐cell light chain—lambda
Kappa/lambda ratio	1.5	Polyclonal (nonclonal) B‐cell population
CD4/CD8 ratio	4.0	Increased CD4 predominance
CD33+/CD34+	40.0%	Myeloid blasts (coexpression of myeloid and stem markers)

*Note:* Immunophenotyping revealed a blast population expressing stem cell markers (CD34, CD117) and myeloid antigens (CD13, CD33, MPO), with partial CD15 expression and low HLA‐DR. The immunophenotype was consistent with acute myeloid leukemia and excluded lymphoid lineage involvement.

**TABLE 3:** Flow cytometry sample viability and cell count.

Parameter	Value
Total cell count	14.7 × 10^6^ cells
Viability	99%

*Note:* Analysis demonstrated a total cell count of 14.7 × 10^6^ cells with 99% viability, confirming high‐quality sampling for flow cytometric analysis.

At presentation, the dermatologic findings were initially nonspecific and raised suspicion for cellulitis, insect bites, bacterial or fungal infection, Sweet syndrome, or a drug reaction, including an immune checkpoint inhibitor–associated eruption, particularly in the setting of recent systemic therapy and immunosuppression. The presence of pruritic plaques with erythema could have been interpreted as infectious or inflammatory, and the accompanying cytopenias were initially attributed to prior chemotherapy exposure. The peripheral smear showing circulating blasts and the confirmatory punch biopsy ultimately established the diagnosis of LC; however, this required consideration of infectious, inflammatory, and paraneoplastic mimics in the differential diagnosis. The diagnostic process was further complicated by the patient’s history of metastatic NSCLC, which raised the possibility of cutaneous metastases, and by overlapping clinical features with drug‐induced rashes. Early skin biopsy with immunophenotyping was therefore critical to avoid misdiagnosis and to expedite the recognition of AML consistent with therapy‐related disease.

### 2.4. Therapeutic Intervention

Empiric ED management included ceftriaxone 1 g IV, potassium repletion (potassium bicarbonate 50 mEq PO and potassium chloride 40 mEq PO plus IV supplementation), hydrocodone/acetaminophen 5 mg PO for pain, and a 1.7 L normal saline bolus. Coverage was escalated to cefepime 2 g IV q8h for neutropenic prophylaxis. AML‐directed supportive care included hydroxyurea 2 g orally twice daily, allopurinol 300 mg orally daily, and transfusion of one unit of packed red blood cells.

Given her advanced age, ECOG performance status of 1, adverse mutational profile, and concurrent metastatic NSCLC, intensive induction chemotherapy was not recommended. Alternatives such as CPX‐351 or azacitidine plus venetoclax were discussed, but she declined further therapy after shared decision‐making based on her preference to avoid additional treatment and to prioritize comfort.

### 2.5. Follow‐Up and Outcomes

She tolerated supportive measures without acute adverse events and transitioned to hospice care, consistent with her goals of care and documented DNR status.

### 2.6. Patient Perspective

The patient emphasized her wish to avoid aggressive chemotherapy and expressed her goals of maintaining comfort and quality of life. Her family shared that the decision to pursue hospice allowed them to focus on her dignity and well‐being during the final phase of her illness. Written informed consent for publication of this case report and the accompanying clinical details and images was obtained directly from the patient.

## 3. Discussion

LC, characterized by the infiltration of leukemic cells into the skin, occurs as the most common extramedullary manifestation in approximately 3%–5% of AML cases [1–3]. However, LC presenting at the time of initial AML diagnosis is uncommon. Based on the 2022 International Consensus Classification (ICC) and the 5th edition of the World Health Organization (WHO‐5) classification systems, this case is consistent with therapy‐related AML, although definitive classification is limited by noninformative conventional cytogenetics. Bone marrow evaluation revealed ≥ 75% myeloblasts with immunophenotypic expression of CD34, CD117, MPO, CD13, and CD33. CD34 and CD117 supported an immature blast population, whereas MPO confirmed myeloid lineage. Low HLA‐DR expression raised consideration of APL, but the absence of Auer rods and negative PML‐RARA FISH argued against that diagnosis. Moreover, Tier 1 pathogenic mutations in DNMT3A, U2AF1, and PPM1D, all of which are associated with secondary AML, were identified [4]. Additionally, the diagnosis of LC, classified under myeloid sarcoma in both ICC and WHO‐5 frameworks, was confirmed by skin biopsy showing dermal infiltration by myeloid blasts expressing CD4, CD43, CD68, and MPO [3]. This established an uncommon presentation of AML with primary cutaneous involvement at initial diagnosis.

t‐AML is a known complication following cytotoxic chemotherapy or radiotherapy, particularly involving alkylating agents or topoisomerase II inhibitors [5]. In this case, the patient received platinum‐doublet chemotherapy (carboplatin/pemetrexed) and docetaxel, which are associated with mutagenic stress and subsequent t‐AML. t‐AML in lung cancer treatment is a relatively rare side effect, accounting for approximately 6% of all t‐AML [6]. The latency period between exposure and onset of t‐AML typically ranges from 2 to 7 years, aligning with this patient’s course, but is longer than many t‐AML in other NSCLC cases [7]. Conventional cytogenetic analysis was noninformative because no metaphase cells were obtained, and AML FISH was normal. Mutations in DNMT3A, U2AF1, and PPM1D are frequently seen in t‐AML and myelodysplastic syndromes (MDSs), suggesting a clonal evolution pathway secondary to treatment‐induced DNA damage, with PPM1D mutations conferring resistance and often present after platinum exposure [4, 5].

Patients with therapy‐related AML often harbor poor prognostic features. These include adverse‐risk cytogenetics, underlying clonal hematopoiesis, and resistance to conventional induction chemotherapy, which all contribute to significantly inferior outcomes compared to de novo AML [7, 8]. For instance, a study analyzing 1133 AML patients found that those with t‐AML had a median overall survival of 13.7 months with intensive therapy, compared to 39.4 months in de novo AML patients [7]. Treatment options available for t‐AML often depend on the functional status of individual patients. In older adults with an ECOG performance status of 0–2, CPX‐351, a dual‐drug liposomal encapsulation of daunorubicin and cytarabine, or azacitidine plus venetoclax is recommended. In younger and more functional patients, induction therapy with a 7 + 3 regimen and allogeneic hematopoietic stem cell transplantation are potential treatment options. Localized radiation therapy can also be used as a palliative measure for symptomatic lesions, alongside other supportive and pain management strategies [2]. In this case, CPX‐351 and azacitidine plus venetoclax were discussed, but the patient elected hospice after shared decision‐making that prioritized comfort in the setting of age, adverse mutational findings, and concurrent metastatic NSCLC.

### 3.1. Strengths and Limitations

Strengths of this case report include comprehensive clinical, histopathologic, immunophenotypic, and molecular characterization, which allowed confirmation of AML with LC, with findings consistent with therapy‐related disease. The integration of imaging, biopsy, flow cytometry, bone marrow findings, and next‐generation sequencing provides a detailed description that adds to the limited literature on this rare presentation. A limitation is the absence of therapeutic outcome data, as the patient elected hospice care and declined chemotherapy. This limited our ability to comment on treatment efficacy, durability of response, or potential complications of therapy. An additional limitation is that conventional cytogenetic analysis was noninformative because no metaphase cells were obtained, limiting definitive classification as therapy‐related AML. Nonetheless, the case underscores the importance of recognizing atypical cutaneous manifestations and highlights decision‐making aligned with patient goals of care.

## 4. Conclusion

This case highlights an uncommon presentation of AML with LC at initial diagnosis in a patient previously treated for NSCLC. Diagnosis was established through histopathologic and immunophenotypic confirmation of myeloid blasts in the skin and bone marrow, supported by pathogenic mutations in DNMT3A, U2AF1, and PPM1D. Conventional cytogenetic analysis was noninformative because no metaphase cells were obtained, while AML FISH was normal and PML‐RARA FISH was negative, making the findings consistent with, rather than definitively diagnostic of, therapy‐related AML. LC, classified as a form of myeloid sarcoma, represents an aggressive extramedullary manifestation of AML and is associated with poor prognosis. Early recognition of atypical cutaneous findings in patients with prior chemotherapy exposure is critical for timely diagnosis and guiding appropriate care decisions.

## Author Contributions

Adam Bowen contributed to conception, data acquisition, manuscript drafting, and revision. Nicholas DiLoreto; Maha Bayya; Robert McDonald; and Daniel Isaac contributed to data interpretation and critical revision of the manuscript.

## Funding

This research received no specific grant from any funding agency in the public, commercial, or not‐for‐profit sectors.

## Disclosure

All authors have read and approved the final version of the manuscript. Adam Bowen had full access to all of the data in this study and takes complete responsibility for the integrity of the data and the accuracy of the data analysis.

## Ethics Statement

Ethical approval is not required for this single‐patient case report.

## Consent

Written informed consent for publication of this case report and accompanying images was obtained from the patient and is available for review by the editor upon request.

## Conflicts of Interest

The authors declare no conflicts of interest.

## Data Availability

The data that support the findings of this study are available from the corresponding author upon reasonable request.
